# Supplementary material for: Cancer-cell intrinsic gene expression signatures overcome intratumoural heterogeneity bias in colorectal cancer patient classification
Source: Nat Commun. 2017 May 31;8:15657. doi: 10.1038/ncomms15657 (PMC5460026; doi:10.1038/ncomms15657)
Supplement: Supplementary Information — Supplementary Figures and Supplementary Table [file ncomms15657-s1.pdf]

Supplementary Figure 1

A

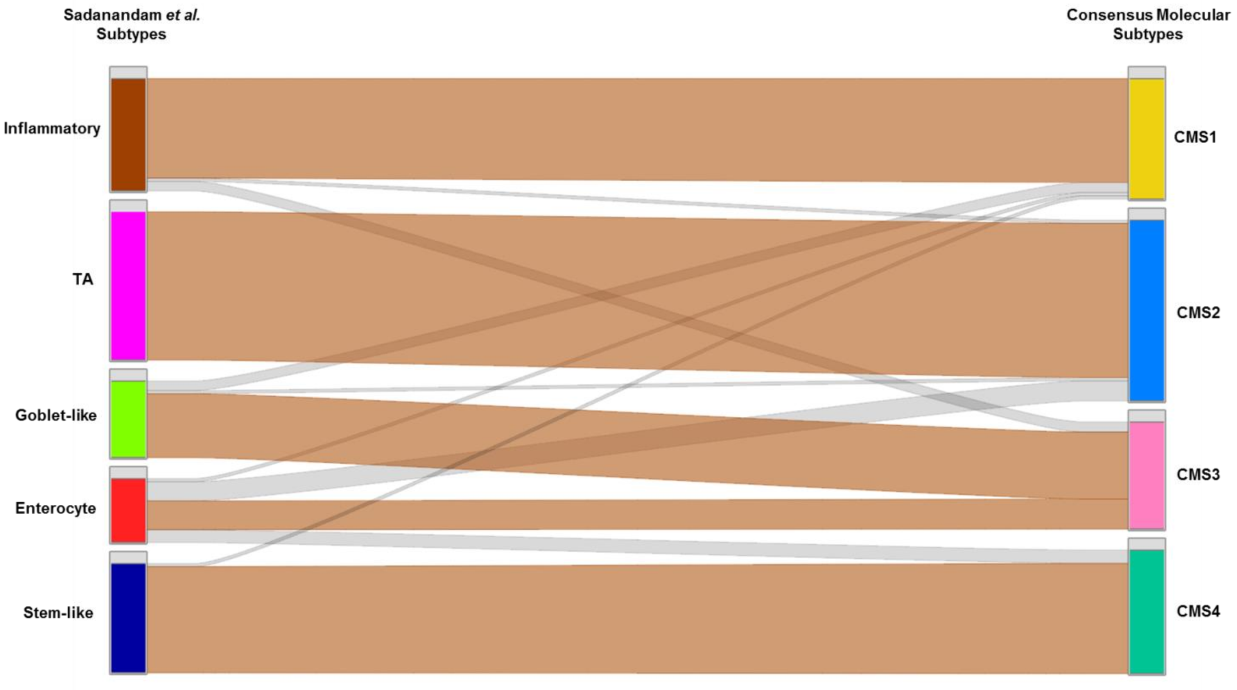

B

	Inflammatory	TA	Goblet-like /Enterocyte	Stem-like
CMS1	86.11		11.11	2.78
CMS2	1.85	85.19	12.96	
CMS3	9.38		90.62	
CMS4			10.81	89.19

C

	CMS1	CMS2	CMS3	CMS4
Inflammatory	88.57	2.86	8.57	
TA		100		
Goblet-like	12.5	4.17	83.33	
Enterocyte	5	30	45	20
Stem-like	2.94			97.04

Supplementary Figure 1: Comparison of CRC sample classification using the *Sadanandam* signature and CMS. A. Caleydo (StratomeX) graphical representation of 159 patients from GSE14333 which could be assigned to a CMS group (using R package 'CMSclassifier') and had previously been classified by *Sadanandam et al.* Orange linker depicts concordant samples between subtypes, grey linker indicates discordant samples between subtypes **B.** Percentage of patients from each CMS group that were assigned to each *Sadanandam* subtype (with "Goblet-like" and "Enterocyte" subtypes being combined into a single group). **C.** Percentage of patients from each *Sadanandam* subtype that were assigned to each CMS group.

Supplementary Figure 2

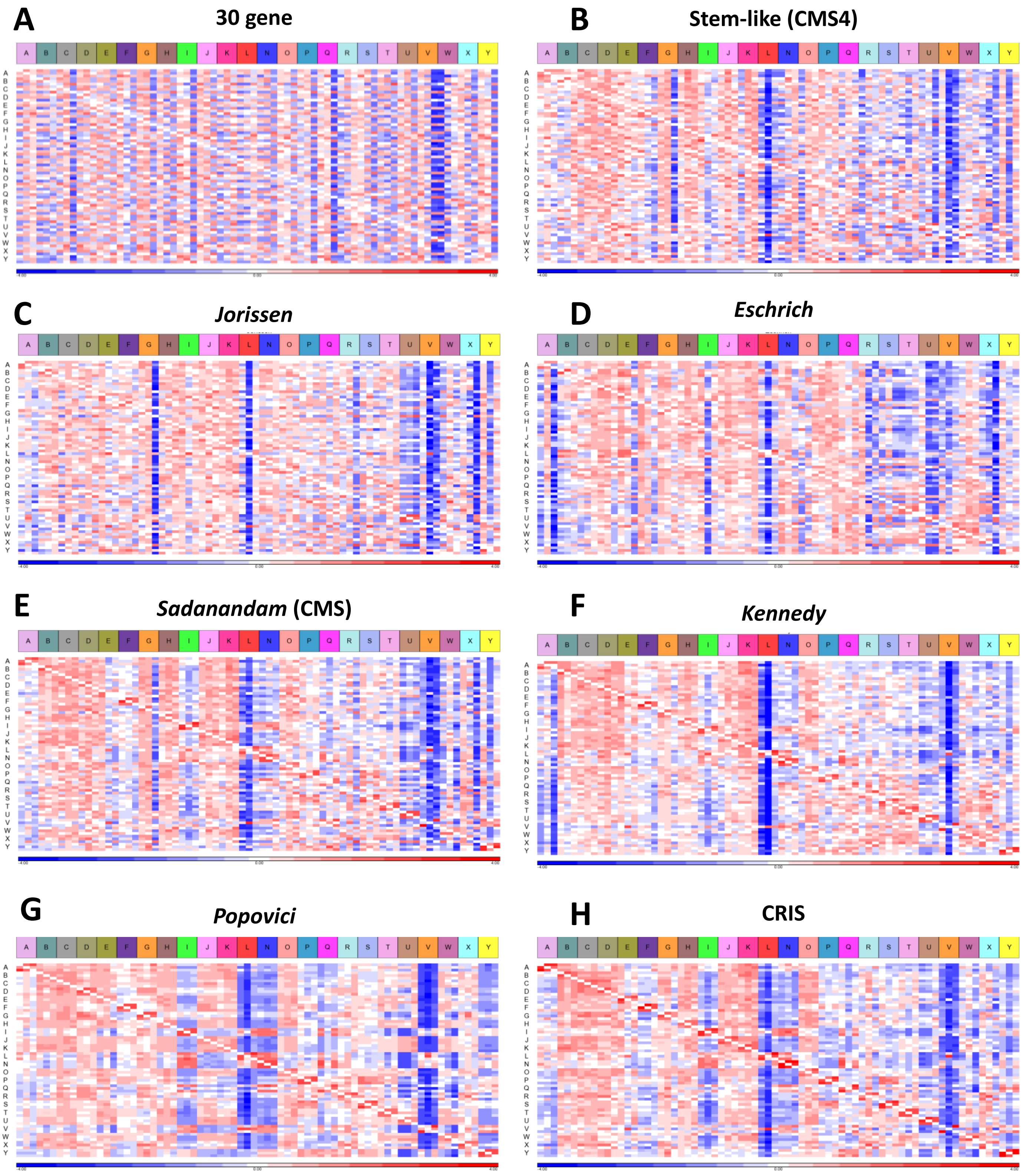

Supplementary Figure 2. A-H. Heatmap representation of Pearson similarity scores according between samples under semi-supervision of the indicated gene expression signatures.

Supplementary Figure 3

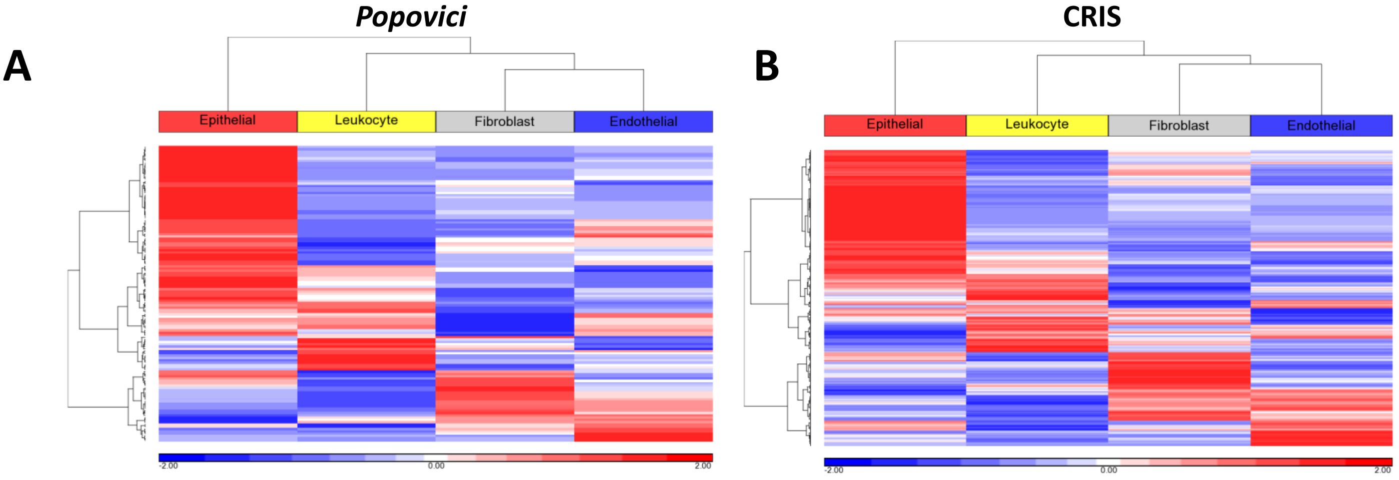

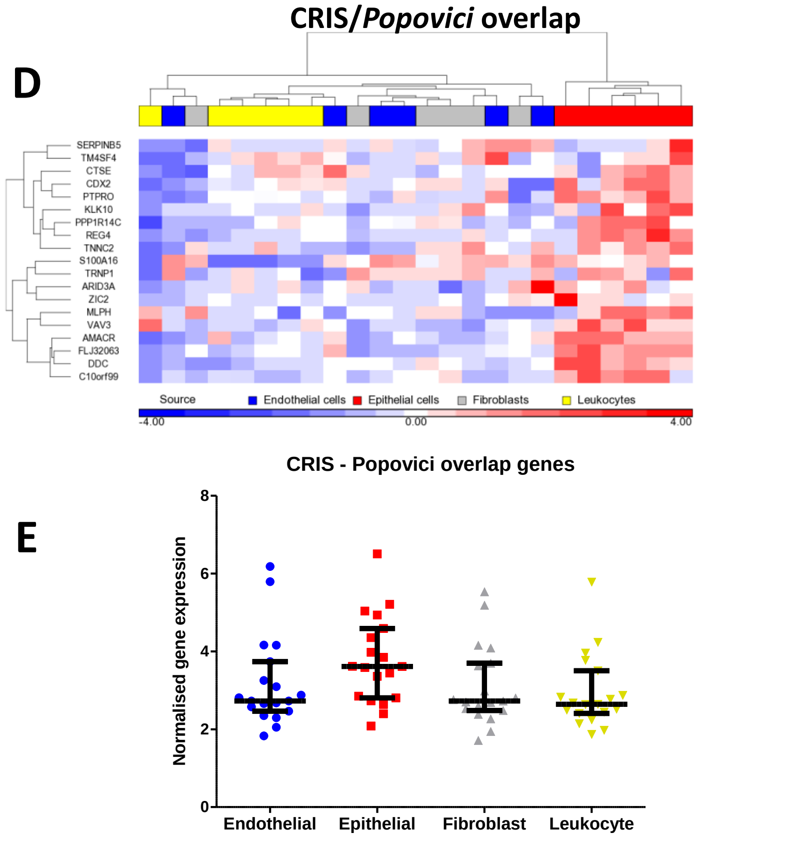

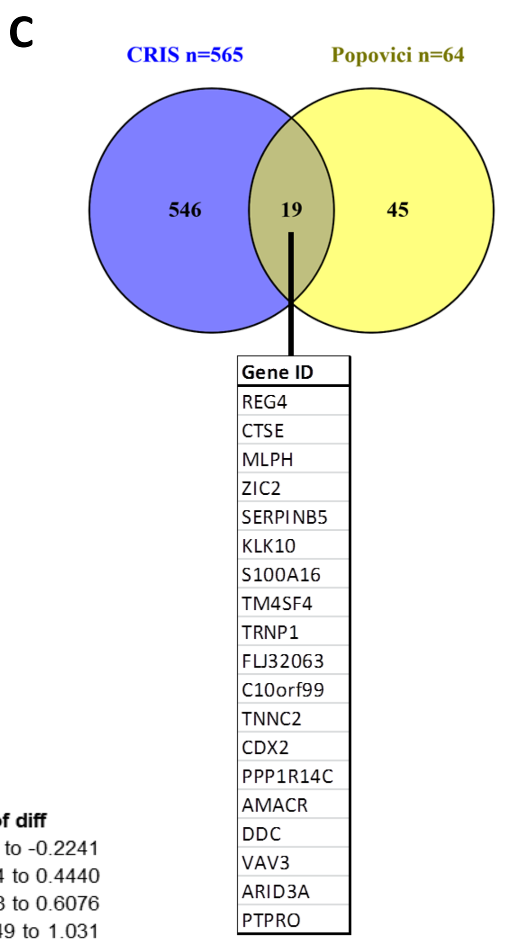

Tukey's Multiple Comparison Test	P < 0.05?	Summary	95% CI of diff
Endothelial vs Epithelial	Yes	***	-0.9505 to -0.2241
Endothelial vs Fibroblast	No	ns	-0.2824 to 0.4440
Endothelial vs Leukocyte	No	ns	-0.1188 to 0.6076
Epithelial vs Fibroblast	Yes	***	0.3049 to 1.031
Epithelial vs Leukocyte	Yes	***	0.4685 to 1.195
Fibroblast vs Leukocyte	No	ns	-0.1996 to 0.5268

Supplementary Figure 3. A + B. Hierarchical clustering of (A) Popovici and (B) CRIS signatures using gene expression profiles from GSE39396. **C.** Venn diagram of Popovici and CRIS genes highlights a 19 gene overlap. **D.** Clustering of 19 overlapping genes using GSE39396 profiles. **E.** Dot plots of data from D confirms significant enrichment of expression for the 19 overlapping genes in epithelial cells. Median and interquartile range are indicated.

Supplementary Figure 4

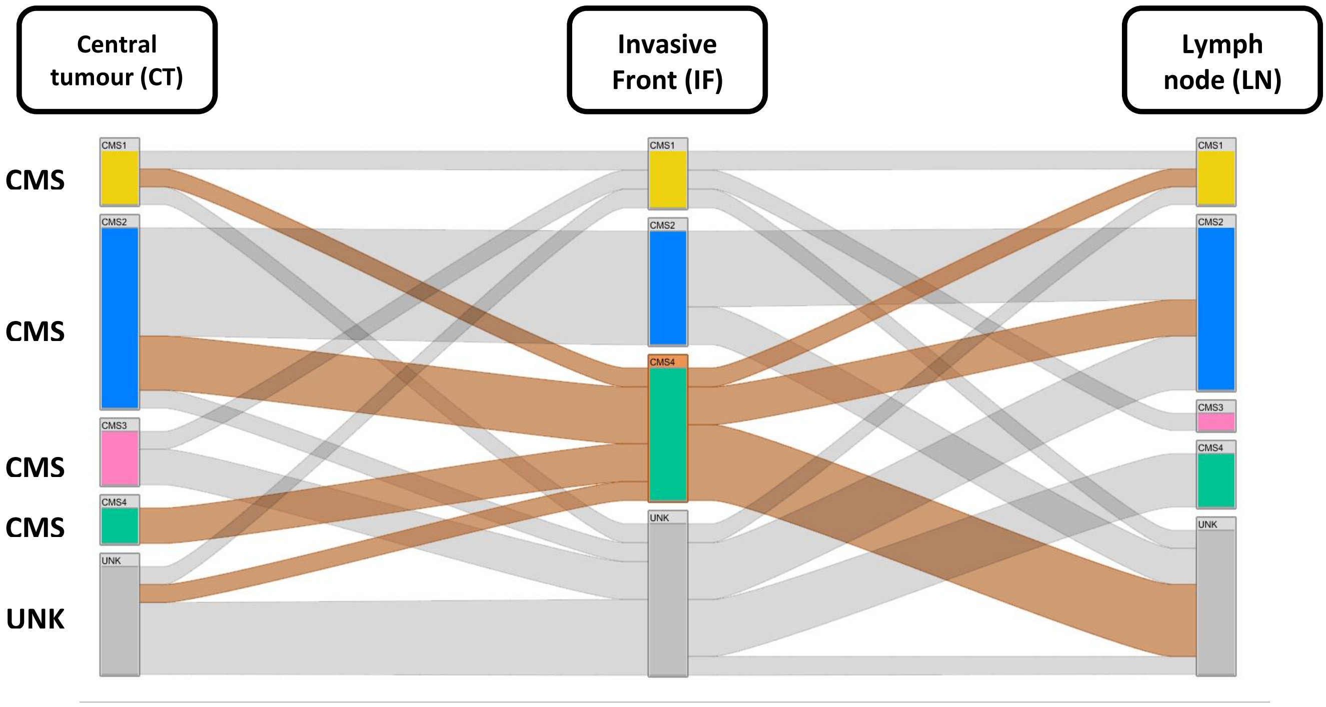

Supplementary Figure 4. Caleydo (StratomeX) graphical representation of the highest predicted CMS score (CMS1-4, UNK=Unknown assignment) for each sample according to region-of-origin (Central, Invasive, Lymph).

Supplementary Figure 5

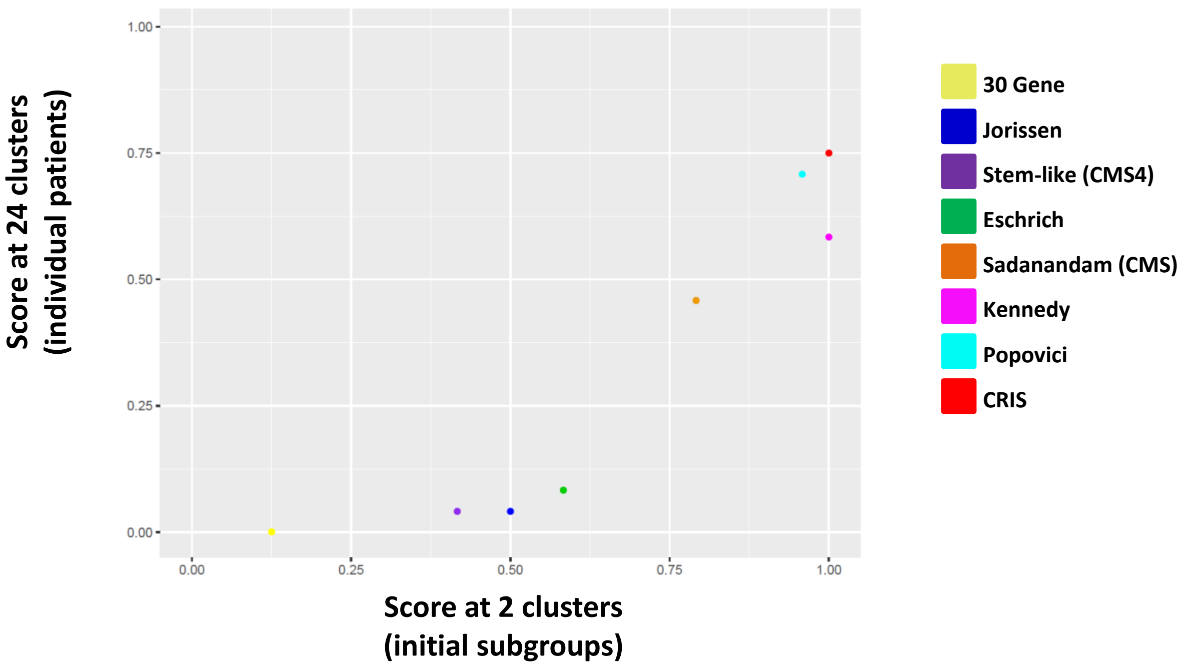

Supplementary Figure 5. Patient group overall ratio expressed as the proportion of patients with all samples clustered together when there are 2 clusters (initial subgroups, x-axis) or 24 clusters (individual patients, y-axis). The color label key for each signature is indicated on the right of each plot.

Supplementary Figure 6

A

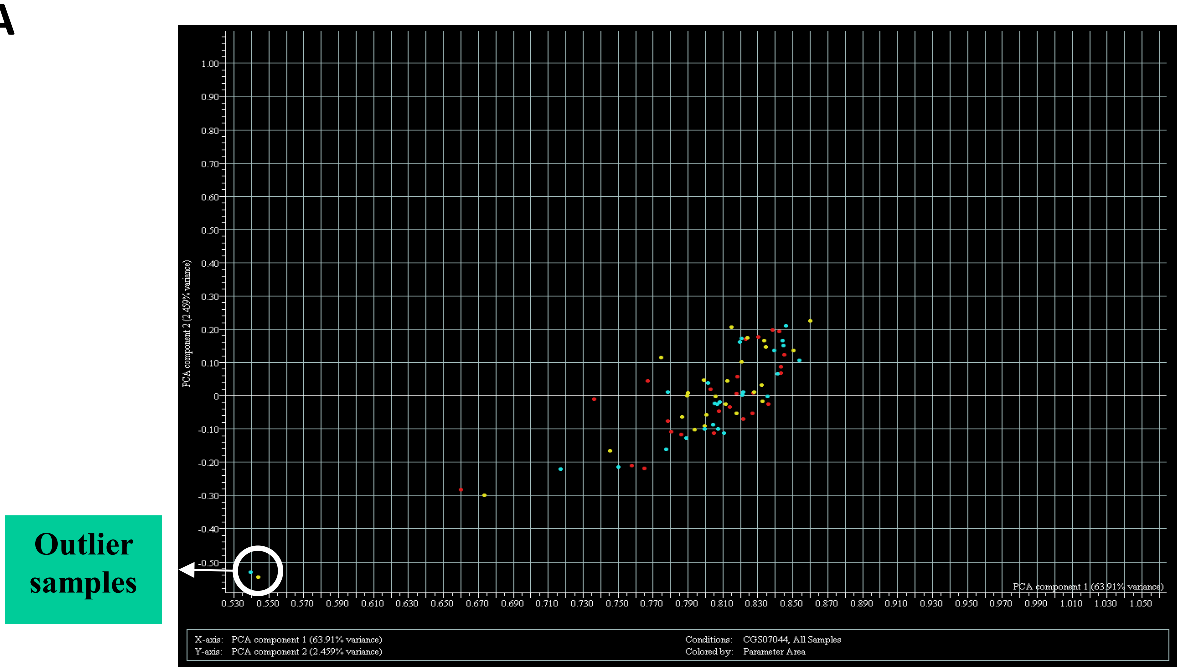

B

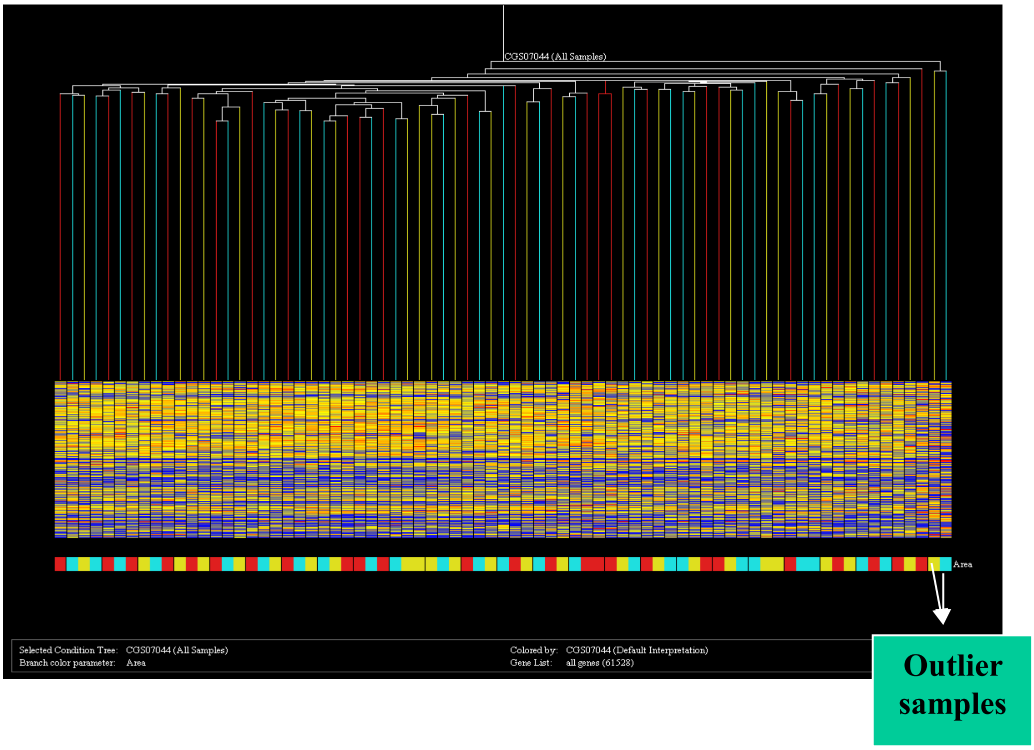

Supplementary Figure 6. A. PCA plot indicating cohort grouping and 2 outlier samples. **B.** Hierarchical cluster of cohort indicating 2 outlier samples. The 2 outliers were removed from subsequent analysis following this initial QC assessment.

Supplementary Table 1

	30 gene	Stem-like CMS4	Jorissen	Eschrich	Sadanandam CMS	Kennedy	Popovici	CRIS
Number of values	24	24	24	24	24	24	24	24
Minimum	-0.8086	-0.3384	-0.6819	-0.4369	-0.3049	-0.1076	-0.05490	-0.2260
25% Percentile	-0.1381	0.1992	0.1496	0.2886	0.3330	0.4539	0.6408	0.4978
Median	0.04056	0.3516	0.4503	0.5291	0.5364	0.5302	0.7344	0.6202
75% Percentile	0.1833	0.4286	0.5366	0.6300	0.5921	0.5922	0.7797	0.6836
Maximum	0.3942	0.5960	0.6438	0.8029	0.6898	0.6911	0.8497	0.7566
Mean	-0.001663	0.2792	0.3105	0.4367	0.4362	0.4881	0.6669	0.5586
Std. Deviation	0.2668	0.2440	0.3550	0.2812	0.2436	0.1804	0.2042	0.2048
Std. Error	0.05446	0.04980	0.07247	0.05740	0.04973	0.03682	0.04168	0.04180
Lower 95% CI of mean	-0.1143	0.1762	0.1606	0.3180	0.3333	0.4119	0.5807	0.4722
Upper 95% CI of mean	0.1110	0.3822	0.4604	0.5555	0.5390	0.5642	0.7531	0.6451
KS normality test								
KS distance	0.1450	0.1762	0.2453	0.1936	0.2492	0.2332	0.2629	0.2239
P value	> 0.10	0.0523	0.0006	0.0204	0.0005	0.0016	0.0002	0.0030
Passed normality test (alpha=0.05)?	Yes	Yes	No	No	No	No	No	No
P value summary	ns	ns	***	*	***	**	***	**
Shapiro-Wilk normality test								
W	0.9023	0.8991	0.8001	0.8607	0.8049	0.7485	0.6921	0.7191
P value	0.0241	0.0206	0.0003	0.0035	0.0004	< 0.0001	< 0.0001	< 0.0001
Passed normality test (alpha=0.05)?	No	No	No	No	No	No	No	No
P value summary	*	*	***	**	***	***	***	***
Coefficient of variation	16038.83%	87.39%	114.35%	64.39%	55.85%	36.96%	30.61%	36.66%
Skewness	-1.307	-1.135	-1.623	-1.598	-1.708	-2.255	-2.540	-2.722
Kurtosis	2.650	0.8650	2.081	2.994	2.835	5.540	6.917	9.194
Sum	-0.03992	6.700	7.452	10.48	10.47	11.71	16.01	13.41

Supplementary Table 1. Table representing normalised Pearson similarity data presented in Figure 5A.
